# LIN-9 Phosphorylation on Threonine-96 Is Required for Transcriptional Activation of LIN-9 Target Genes and Promotes Cell Cycle Progression

**DOI:** 10.1371/journal.pone.0087620

**Published:** 2014-01-27

**Authors:** Frank Eckerdt, Mathew Perez-Neut, Oscar R Colamonici

**Affiliations:** Department of Pharmacology, University of Illinois at Chicago, College of Medicine, Chicago, Illinois, United States of America; Rush University Medical Center, United States of America

## Abstract

Cell cycle transitions are governed by the timely expression of cyclins, the activating subunits of Cyclin-dependent kinases (Cdks), which are responsible for the inactivation of the pocket proteins. Overexpression of cyclins promotes cell proliferation and cancer. Therefore, it is important to understand the mechanisms by which cyclins regulate the expression of cell cycle promoting genes including subsequent cyclins. LIN-9 and the pocket proteins p107 and p130 are members of the DREAM complex that in G0 represses cell cycle genes. Interestingly, little is know about the regulation and function of LIN-9 after phosphorylation of p107,p130 by Cyclin D/Cdk4 disassembles the DREAM complex in early G1. In this report, we demonstrate that cyclin E1/Cdk3 phosphorylates LIN-9 on Thr-96. Mutating Thr-96 to alanine inhibits activation of cyclins A2 and B1 promoters, whereas a phosphomimetic Asp mutant strongly activates their promoters and triggers accelerated entry into G2/M phase in 293T cells. Taken together, our data suggest a novel role for cyclin E1 beyond G1/S and into S/G2 phase, most likely by inducing the expression of subsequent cyclins A2 and B1 through LIN-9.

## Introduction

Cell cycle transitions are tightly regulated by the timely expression and degradation of cyclins, the regulatory subunits of cyclin-dependent kinases (Cdks). E-type cyclins are exclusively available at early stages of DNA synthesis and a large body of evidence suggests that they are essential to drive G1/S transition [1]. E-type cyclins are found overexpressed in a variety of human cancers and are believed to contribute to oncogenesis [2]. However, they are largely dispensable in normal somatic cells and for mouse development [3], thus making them an attractive target for cancer therapy. As E-type cyclins are dispensable for normal somatic cells but essential for tumor cell proliferation, it is important to understand how E-type cyclins promote cell cycle progression.

A key role of cyclin E1 is the binding and activation of Cdk2, thereby promoting G1/S transition and centrosome duplication [2]. In addition to Cdk2, Cyclin E1 can activate Cdk3, which is structurally closely related to Cdk2 [4]–[8]. Early reports indicate that Cdk3 plays a unique role in the G1/S transition. For instance, dominant-negative Cdk3-DN induces a G1/S arrest that cannot be overcome by the expression of SV40, while a similar arrest produced by Cdk2-DN can be rescued by SV-40 expression but not by Cdk3 [7]. More importantly, the G1 arrest induced by Cdk3-DN can be rescued by simultaneous expression of Cdk3, but not Cdk2 [8]. These observations demonstrate that Cdk3 exerts unique functions in G1/S phase that cannot be compensated by Cdk2. Cdk3 and other G1 Cdks are responsible for the phosphorylation and inactivation of the pocket proteins retinoblastoma (pRB), p107 and p130 [9], which release the inhibition that pRB/E2F1-3 and p107,p130/E2F4-5 exert on many genes required for S-phase entry [10]–[13]. Additionally, E2Fs are also necessary for the control of mitotic genes. For example, the transcriptional activation of cyclins A and B, and Cdk1 require the coordinated action of E2F1-3a and other transcription factors such as B-Myb [14]. B-Myb is part of a complex that has been termed dREAM (drosophila RB, E2F And Myb) after a similar complex originally described in *Drosophila* [15]–[17] and has been linked to the cell cycle regulation in G0 and S phases. A similar complex termed DRM or MuvB has been described in *C. elegans*, but unlike the dREAM complex, there is no known homolog of B-Myb in *C. elegans*. In *Drosophila*, the Myb-interacting protein complex is required for DNA replication of specific developmental genes (chorion), and in the absence of Myb there is no initiation of replication even after loading of preRC [15].

Although the mammalian counterpart of the dREAM complex contains similar proteins, there are critical differences in complex formation and function among species. In mammalian cells, the counterpart of the dREAM complex is only found in quiescent cells, while a complex that includes LIN-9 (Mip130), LIN-37 (Mip40), LIN-52; and LIN-54 (Mip120), termed MCC/LINC (Mip Core Complex, LIN Complex, Multivulva Complex), is stable throughout the cell cycle and specifically binds to p107,p130/E2F4 in G0 and B-Myb in S-phase [18]–[21]. The interaction of the MCC/LINC complex with B-Myb in S-phase is critical for the induction of G2/M genes [19]–[22]. Recently, it has been shown that the induction of G2/M genes also requires the interaction of this complex with FoxM1 [23]. Interestingly, little is known about post-translational modifications, such as phosphorylation, that may regulate the function of the MCC/LINC. It has been reported that phosphorylation of LIN-52 was important for the regulation of quiescence and that LIN-9 was phosphorylated in vivo [24]. However, the biological role of this post-translational modification of LIN-9 was not investigated. In this report, we investigate the role of LIN-9 phosphorylation in the transcriptional activation of cell cycle genes. We found that LIN-9 is phosphorylated in a cell cycle dependent manner in cycling cells. We identify Cdk3/cyclin E1 as a novel LIN-9 targeting kinase and provide strong evidence for this phosphorylation being essential for promoting cell cycle progression.

## Results

### LIN-9 is phosphorylated by and associates with cyclin E/Cdk3

**Figure 1:**
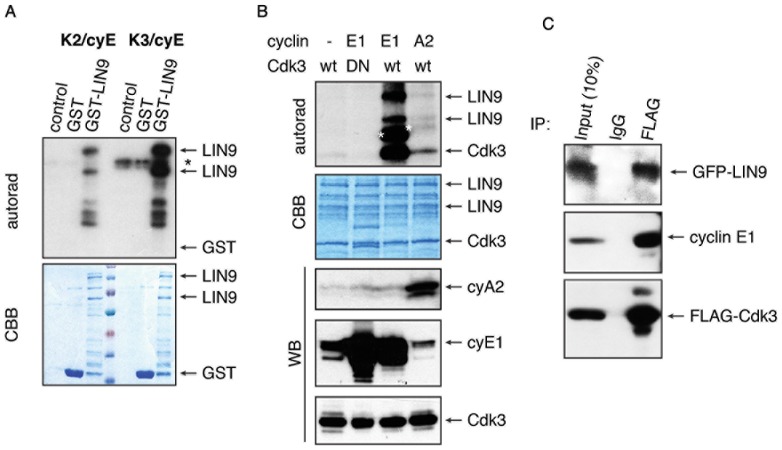
LIN-9 is phosphorylated by and is found in a trimeric complex with Cdk3/cyclin E1. (A) GST-LIN-9 expressed in bacteria was subjected to in vitro kinase assays using indicated kinase complexes purchased from ProQinase. Asterisk indicates GST-cyclin autophosphorylation. The lower panel shows Coomassie blue staining of the same gel. (B) GST-LIN-9 was subjected to in vitro kinase assays using Flag-Cdk3 kinase complexes (Flag-IP) from human 293Tcells. Autoradiography visualizes incorporation of phospholabel and asterisks (white, placed to the left of the corresponding band) indicate autophosphorylation of cyclin E1 and cyclin A2, respectively (upper panel). Coomassie Brillant Blue (CBB) staining depicts loading of GST-LIN-9 and Flag-Cdk3 (second panel). Ten percent of IP material was subjected to Western blot to visualize amounts of co-precipitated cyclin A2 (third panel), cyclin E1 (fourth panel) and Flag-Cdk3 (lower panel). (C) 293T cells were cotransfected with Flag-Cdk3 and GFP-LIN-9, followed by Flag-IP. IP material was subjected to Western blots using LIN-9 antibody (upper panel), cyclin E1 antibody (middle panel) and Flag-antibody (lower panel).

In cycling cells, LIN-9 promotes the transcription of cell cycle genes such as cyclins A2 and B1, thereby facilitating Cdk activation, and promoting cell cycle transitions [19], [21]. To test whether LIN-9 is phosphorylated by cell cycle protein kinases, we subjected GST-LIN-9 expressed in bacteria to *in vitro* kinase assays using a panel of activated protein kinases (Cdk4/cycD3, Cdk4/cycD1, Cdk6/cycD1, Cdk3/cycE1, Cdk2/cycE1, Cdk1/cycA2, Cdk1/cycB1, Cdk9,cycT, Cdk7/cycH, Cdk5,p35, Cdk5/p25). From this panel, Cdk3/cyclin E1 and to a lesser extend Cdk2/cyclin E1, showed strong kinase activity towards GST-LIN-9 (Fig. 1A and data not shown). Given this observation, we sought to investigate whether Cdk3 can phosphorylate LIN-9 under more physiological conditions. As Cdk3 associates with cyclins E and A in proliferating cells [25], we co-transfected 293T cells with Flag-Cdk3 and untagged cyclin E1 or cyclin A2, immunoprecipitated Flag-Cdk3 and associated cyclins using an anti-Flag antibody, and subjected the immunoprecipitated material to in vitro kinase assays using GST-LIN-9 as a substrate. When Cdk3 was transfected alone, weak autophosphorylation of Cdk3 was detectable but no LIN-9 phosphorylation was evident (Fig. 1B, upper panel, lane 1), consistent with the notion that Cdks require cyclins for activation. However, when Cdk3 was co-expressed with cyclin E1, phosphorylation of LIN-9, cyclin E1, and autophosphorylation of Cdk3, was very strong (Fig. 1B, upper panel, lane 3) indicating that cyclin E1 is a potent activator of Cdk3 and the Cdk3/cyclin E1 complex targets LIN-9 for phosphorylation. Cdk3 co-expressed with cyclin A2 showed kinase activity towards LIN-9, albeit to a much lesser extend (Fig. 1B, upper panel, lane 4). Importantly, when cyclin E1 was co-expressed with Flag-Cdk3-DN (a kinase dead derivative in which Asp-145 was replaced by Asn) no phosphorylation was evident, demonstrating the specificity of the assay (Fig. 1B, upper panel, lane 2). We confirmed that similar levels of Flag-Cdk3 were precipitated and associated with the respective co-expressed cyclin by subjecting 10% of IP material to Western blots using antibodies for Flag (detecting Flag-Cdk3), cyclin E1 and cyclin A2 (Fig. 1B, WB lower panels). Coomassie brilliant blue staining of the kinase assay confirmed equal loading of GST-LIN-9 (Fig. 1B, second panel, CBB). These results confirm our previous in vitro findings of LIN-9 being phosphorylated predominantly by Cdk3. Additionally, they demonstrate that cyclin E1/Cdk3 is the major cyclin/Cdk complex phosphorylating LIN-9 because when this Cdk is associated with other cyclins, i.e. cyclin A2, phosphorylation of LIN-9 was very weak.

As Cdk3 in complex with cyclin E1 is the major kinase phosphorylating LIN-9, we tested whether all these proteins can be found in a trimeric complex in cells. Thus, we co-transfected 293T cells with Flag-Cdk3 and GFP-LIN-9 and subjected cell extracts to immunoprecipitations with an anti-Flag antibody followed by Western blot. Figure 1C demonstrates that Flag-Cdk3 immunoprecipitates (lower panel) contained substantial amounts of both endogenous cyclin E (middle panel) and GFP-LIN-9 (upper panel). Taken together, these observations indicate that Cdk3/cyclin E1 is the major kinase phosphorylating LIN-9 during the cell cycle and that LIN-9, Cdk3 and cyclin E1 can be found in a trimeric complex in human cells.

### Thr-96 in LIN-9 is the major phosphorylation site for Cdk3/cyclin E1

**Figure 2:**
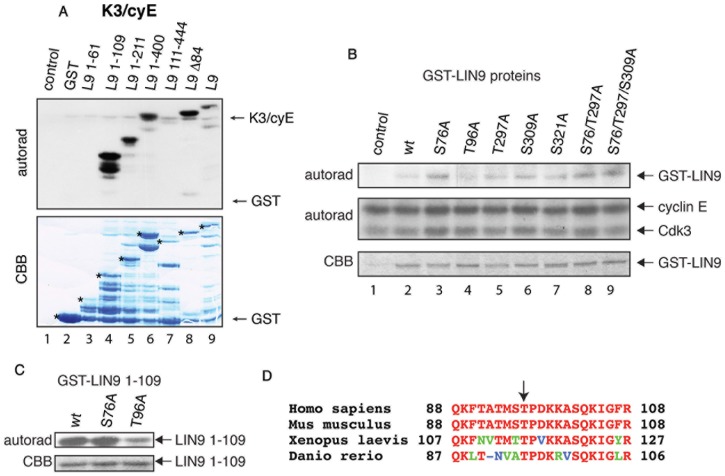
Thr-96 is the major phosphorylation site in LIN-9 for Cdk3/cyclin E1. Indicated GST-LIN-9 deletion constructs were expressed in bacteria, purified, and subjected to in vitro kinase assays using Flag-IPs from 293T cells cotransfected with Flag-Cdk3 and untagged cyclin E1. Autoradiogram shows phosphorylation of recombinant LIN-9 proteins (upper panel). CBB staining of the same gel was used to visualize the amounts of GST-LIN-9 constructs in the reaction (lower panel) Asterisks indicate the respective GST-LIN-9 constructs. (B) Indicated GST-LIN-9 full-length mutants expressed in bacteria were subjected to in vitro kinase assays using Flag-Cdk3/cyclin E precipitated from 293T cells, transfected with FLAG-Cdk3 and cyclin E1, via Flag-antibody as a kinase. LIN-9 phosphorylation (upper panel), Flag-Cdk3 and cyclin E1 autophosphorylation (middle panel) were monitored by autoradiography. Loading of GST-LIN-9 mutants was determined by CBB staining of the same gel (lower panel). (C) GST-LIN-9-1-109 and its mutants (S76A and T96A) were subjected to kinase assays as in B. GST-LIN-9 phosphorylation was again detected by autoradiography (upper panel) and equal loading was confirmed by CBB staining of the same gel (lower panel). (D) Alignment of LIN-9 amino acid residues surrounding Thr-96 among species. Identical residues are depicted in red, strongly similar residues are depicted in green, and non-similar residues are depicted in blue. Arrow indicates position of Thr-96. Accession numbers of aligned amino acid sequences are: Homo sapiens: AAV41873.1; Mus musculus: NP_001096652.1; Xenopus laevis: NP_001121233.1 and Danio rerio: NP_001038411.1.

To narrow down the Cdk3/cyclin E1 phosphorylation site in LIN-9 we first produced deletion constructs of GST-LIN-9 in bacteria and subjected them to *in vitro* kinase assays using Flag-Cdk3/cyclin E1 complexes immunoprecipitated from 293T cells as a source for kinase activity. A truncated form of LIN-9 encoding residues 1-61 (amino acids sequence as reported in [26], although expressed at lower levels than other GST-LIN-9, was not phosphorylated at all (Fig. 2A, lane 3, LIN-9 1–61), but a fragment containing amino acids 1-109 showed strong phosphorylation by Cdk3/Cyclin E1 (lane 4), suggesting that LIN-9 is phosphorylated by this kinase complex at a region between amino acids (aa) 61 and 109. Consistently, LIN-9 deletion constructs containing residues 1–211 (lane 5) and 1–400 (lane 6) were phosphorylated, whereas a construct encompassing aa 111–444 was not (lane 7). Additionally, a construct encompassing aa 85–542 (L9 Δ84) was still a good substrate (lane 8). This indicates that the major phosphorylation site for Cdk3/cyclin E1 in LIN-9 resides between aa 85–109.

Cdk/cyclins are proline directed kinases targeting Serine/Threonine-Proline (S/T-P) motives. As an additional approach we mutated every potential Cdk site (S/T-P) in LIN-9 to Alanine (A). Potential Cdk sites were S76, T96, T297, S309 and S321. We substituted all these potential Cdk sites in LIN-9 to A, expressed them as GST-LIN-9 proteins in bacteria and subjected them to *in vitro* kinase assays using Flag-Cdk3/cyclin E immunoprecipitated from human cells as kinase source. Of all GST-LIN-9 mutants, GST-LIN-9-T96A showed the weakest incorporation of radiolabel (Fig. 2B, lane 4), consistent with the notion that the major phosphorylation site resides between aa 85-109. However, other LIN-9 mutants, in particular T297A, exhibited reduced phosphorylation as well (Fig. 2B, lane 5), indicating that LIN-9 could be phosphorylated by Cdk3/cyclin E on additional sites, such as Thr-297. Thus, we generated double and triple mutants. As demonstrated in Fig. 2B, the double mutant GST-LIN-9^S76/T297A^ (lane 8) and the triple mutant GST-LIN-9^S76/T297/S309A^ (lane 9) were readily phosphorylated, suggesting that Thr-96, is the major phosphorylation site for Cdk3/cyclinE. It is worth mentioning that phosphorylation of wild type GST-LIN-9 was weaker in this assay due to lower expression of Cdk3 and, as evident from figure 2A, the deletion constructs are better substrates than the full-length LIN-9 protein.

**Figure 3:**
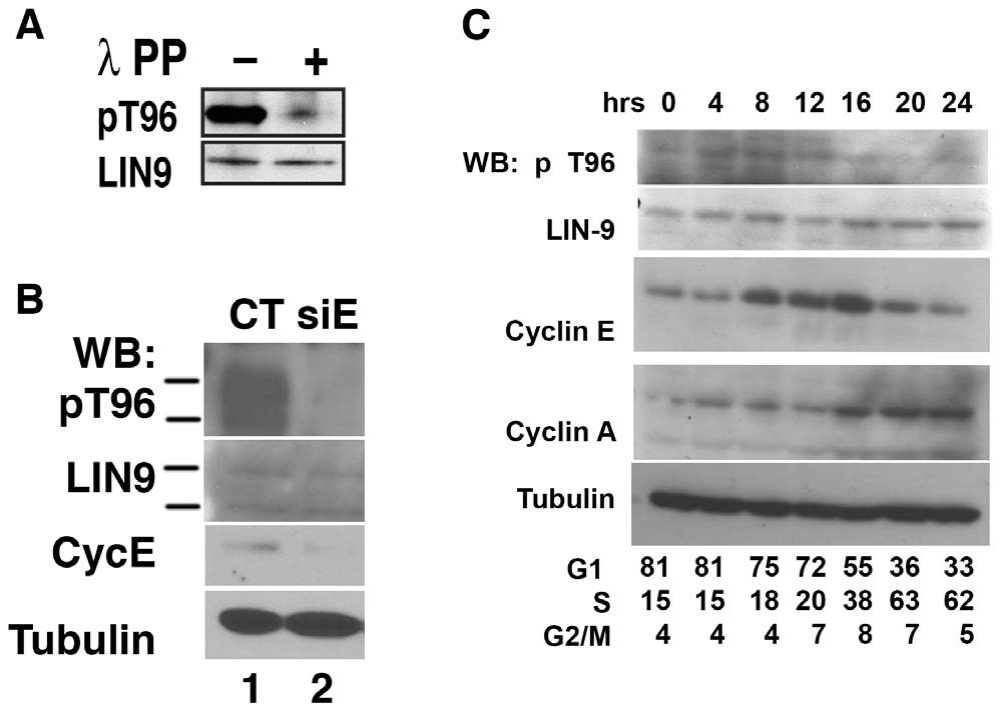
cyclin E is essential for phosphorylation of Thr-96 in LIN-9 in vivo in primary cells. (A) p-LIN-9^Thr-96^ antibody is specific. 293T cells were cotransfected with plasmids encoding GFP-LIN-9, FLAG-Cdk3 and cyclin E, and cell extracts were subjected to immunoprecipitation using a monoclonal LIN-9 antibody. IP material was split into two, and incubated for 1 hour at 30°C with or without lambda phosphatase. Samples were subjected to Western Blot using our p-LIN-9^Thr-96^ antibody (upper panel). Equal loading of LIN-9 was confirmed by incubating the same membrane with a rabbit antibody against LIN-9 (lower panel). (B) HUVECs were transfected with siRNA control or siRNA directed against Cyclin E1 (Santa Cruz) using Dharmafect 4 (Dharmacon) and harvested after 48 hours. Western Blots was performed with the indicated antibodies. (C) T98G cells were starved in serum-free medium for 36 hours followed by the addition of growth medium (supplemented with 10% serum). Cells were harvested by tripsynization at the indicated time after the addition of serum and use for Western blot or FACS analysis. Western blots were performed as described in Methods.

To further corroborate our finding that the Cdk3/cyclin E phosphorylation site in LIN-9 resides between aa 85 and 109 (see Fig 3A) we used a deletion construct (LIN-9 1–109) that was readily phosphorylated by Cdk3/cyclin E1 (see Fig. 2A) and introduced mutations S76A and T96A. Of both constructs, only LIN-9 1–109^T96A^ showed a substantial reduction in phosphorylation (Fig. 2C). Taken together, our extensive analysis of LIN-9 deletion constructs and LIN-9 mutants suggest that the major phosphorylation site for Cdk3/cyclin E in LIN-9 is Thr-96. Alignment of the aa sequence surrounding Thr-96 revealed that the Cdk consensus site T96-P97 is present in vertebrates from fish to human (Fig. 2D). Given the high conservation of this motif, it is likely that the phosphorylation of Thr-96 observed in human cells represents an event highly conserved among vertebrates.

### Knockdown of Cyclin E impairs phosphorylation of Thr-96 of LIN-9 in HUVECs

To test whether Thr-96 in LIN-9 is phosphorylated by Cdk3/cyclin E in vivo, we produced a phoshpho-specific antibody, recognizing LIN-9 only when phosphorylated on Thr-96 (p-LIN-9^Thr-96^). First we tested the p-LIN-9^Thr-96^ antibody for specificity. As LIN-9 phosphorylation is most prominent when cells are overexpressing Cdk3 and cyclin E (see figure 1B), we cotransfected 293T cells with plasmids encoding GFP-LIN-9, FLAG-Cdk3 and cyclin E, to ensure efficient phosphorylation of Thr-96 in LIN-9. Resulting cell extracts were subjected to immonoprecipitation using our own mouse monoclonal LIN-9 antibody. IP material was split into two, and incubated for 1 hour at 30°C with, or without, lambda phosphatase. Samples were subjected to Western Blot using our p-LIN-9^Thr-96^ antibody. Our p-LIN9^Thr-96^ antibody detected a strong signal from the IP material that was incubated without lambda phosphatase (Fig. 3A, first lane), whereas the signal from the sample that was incubated with lambda phosphatase was greatly reduced (Fig. 3A, second lane). This indicates that the phosphatase efficiently dephosphorylated LIN-9 on Thr-96 and that the p-LIN-9^Thr-96^ antibody is specific. Equal loading of LIN-9 was confirmed by incubating the same membrane with our rabbit antibody against LIN-9 (Fig. 3A, lower panel). We also confirmed the specificity of the p-Thr-96 antibody by using GST-LIN-9 produced in bacteria, phosphorylated by FLAG-IP material from cells transfected with FLAG-Cdk3/cyclin E in vitro using FLAG-Cdk3-DN/cyclin E as a negative control (data not shown). These data suggest, that our p-LIN-9^Thr-96^ antibody specifically recognizes LIN-9 when phosphorylated by cyclin E/Cdk3 on Thr-96. Next we sought to investigate whether cyclin E is essential for phosphorylation of Thr-96 of endogenous LIN-9 in primary cells in vivo. Therefore, we transfected primary Human Umbilical Vein Endothelial Cells (HUVEC) with siRNA targeting cyclin E and monitored phosphorylation of LIN-9 on Thr-96 using our p-LIN-9^Thr-96^ antibody. In cells transfected with control siRNA, pThr-96 of LIN-9 was readily detectable (Fig. 3B, upper panel, first lane). However, in cells transfected with siRNA against cyclin E, phospho-Thr-96 was undetectable (Fig. 3B, upper panel, second lane) indicating that knockdown of cyclin E greatly impairs phosphorylation of LIN-9 on Thr-96 in vivo in primary cells. Western Blot for LIN-9 (second panel), cyclin E (third panel) and tubulin (fourth panel) confirmed equal loading and efficient knockdown of cyclin E in this experiment. In summary, the data obtained by our p-LIN-9^Thr-96^ antibody indicate that LIN-9 is phosphorylated on Thr-96 in vivo and that cyclin E is essential for mediating this phosphorylation in primary cells.

We next performed cell cycle synchronization experiments using T98G cells followed by immunoblotting with the p-LIN-9^Thr-96^ antibody to determine the cell cycle distribution of p-LIN-9^Thr-96^ and to assess the phosphorylation of LIN-9 in tumor cells. Thr-96 phosphorylation of LIN-9 was detected at low levels in G0, and peaked at 8 and 12 hours, a time when cyclin E expression also increased (Fig. 3C). FACS analysis revealed that the subsequent decrease in LIN-9 phosphorylation observed after 12 hours coincides with cells entering S-phase. Interestingly, after 12 hours, a time when phosphorylation of LIN-9 on Thr-96 decreases, expression of cyclin A is induced. Thus, LIN-9 phosphorylation precedes the rise in cyclin E expression, which in turn is followed by cyclin A expression. These data suggest that phosphorylation of LIN-9 is an early G1 event that is likely driven by Cyclin E/Cdk3. It is not surprising that Cyclin E expression continuous to increase beyond the G1/S boundary since it has been proposed that the activation of Cdk2 by this cyclin occurs after the activation of Cyclin E/Cdk3 closer to the G1/S boundary and can still be detected in early S–phase [7], [8].

### Mutations of Thr-96 of LIN-9 affect the activation of specific promoters

**Figure 4:**
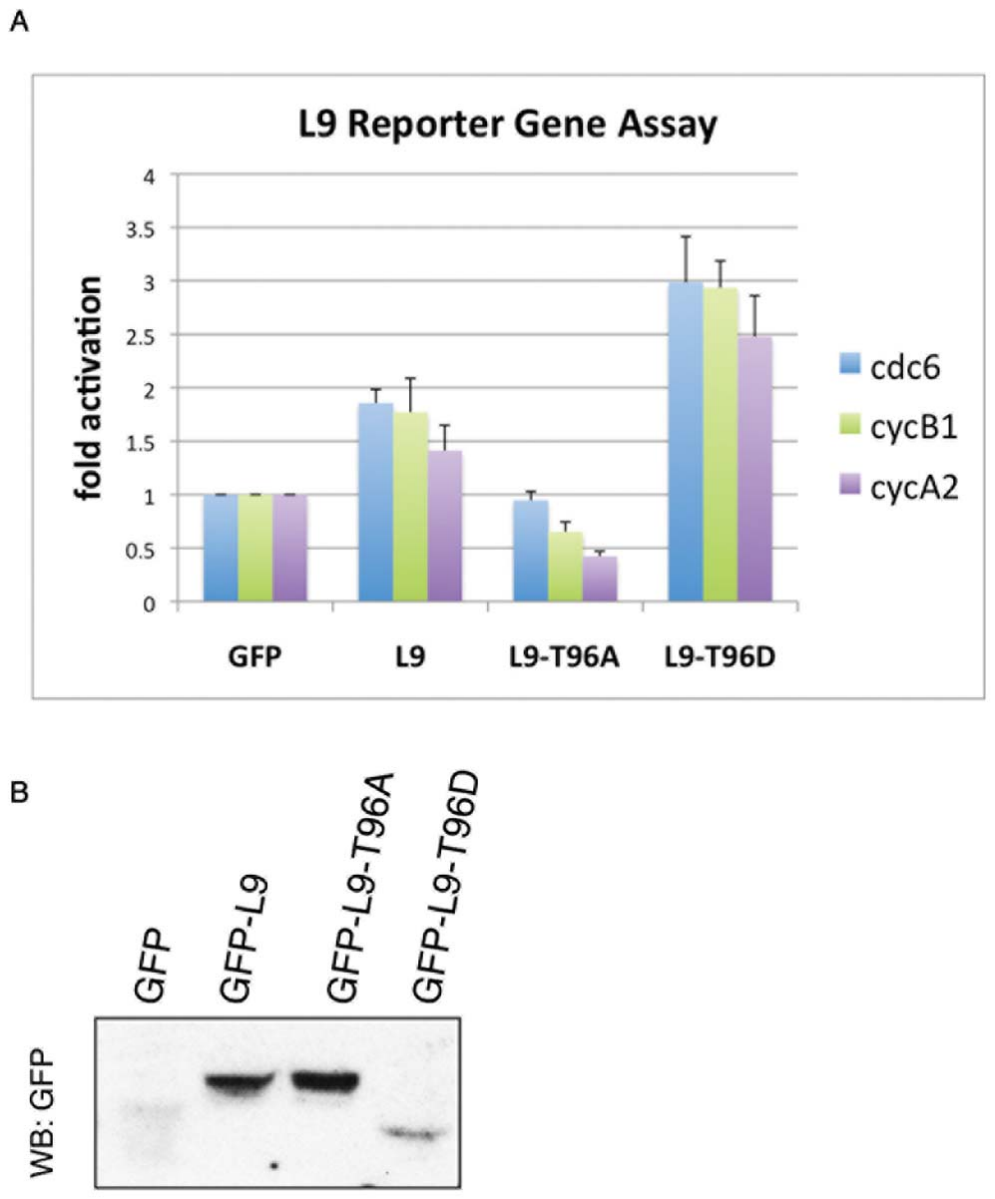
Phosphorylation of Thr-96 in LIN-9 is essential for transcriptional activation of cell cycle genes. (A) Indicated GFP-LIN-9 constructs were cotransfected into 293T cells, together with luciferase under the control of the promoters of cdc6, cyclin B1 or cyclin A2. Cell lysates were subjected to reporter gene assays using Promega Glo (Promega). Experiment was performed in triplicates. For the cdc6 promoter: n = 4, for the cyclin A and cyclin B promoters: n = 2; *p<0.05, **p<0.005, ***p<0.0005 (B) One of the three samples used in A was subjected to Western blot, using GFP antibody to detect expression of GFP-LIN-9 constructs. GFP-LIN-9^T96D^ migrates faster and is always expressed at lower levels than GST-LIN-9 wild type and T96A.

In cycling cells, LIN-9 is known to induce activation of cyclins A2 and B1 promoters [19], [21], [23], [27]. Additionally, we have demonstrated that LIN-9 also binds to the cdc6 promoter, which plays a role in senescence and cell cycle progression [28]. To test whether phosphorylation of LIN-9 on Thr-96 by Cdk3/cyclin E is important for LIN-9-mediated cell cycle progression by inducing transcription of cell cycle genes, we investigated whether mutants that cannot be phosphorylated (LIN-9 T96A) or mimic phosphorylation (T96D) are capable of inducing activation of cell cycle genes. To this end, we performed reporter gene assays using the promoters of cdc6, cyclin A2 and cyclin B1. Wild-type LIN-9 readily induced activation of cdc6, cyclin B1 and cyclin A2 promoters, but non-phosphorylatable LIN-9^T96A^ failed to do so. Moreover, in the case of the cyclin B1 and A2 luciferase reporters, this construct resulted in promoter activation below control levels, suggesting that the T96A mutation has a dominant negative effect by interfering with endogenous LIN-9 function (Fig. 4A). Importantly, LIN-9^T96D^, mimicking phosphorylation on Thr-96, was much more potent in inducing cdc6, cyclin B1 and cyclin A2 promoter activation than its wild-type counterpart. Taken together, these results strongly suggest that phosphorylation of LIN-9 on Thr-96 is essential for inducing the transcriptional activation of cell cycle genes such as cyclins A2, B1 and cdc6. Noteworthy, the LIN-9-T96D mutant was expressed at much lower levels and migrated faster than both Flag-tagged and GFP-tagged forms in all experiments (Fig. 4B and data not shown). Nevertheless, given the low abundance of LIN-9-T96D and the striking enhancement observed for promoter activation of cdc6, cyclin B1 and cyclin A2, it is likely that phosphorylation of LIN-9 on Thr-96 by Cdk3/cyclin E is a key event, triggering cell cycle progression by activating cdc6, cyclins A2 and B1 and possibly other cell cycle genes.

### Thr-96 phosphorylation of LIN9 is important for promotion of S to G2/M transition of the cell cycle

**Figure 5:**
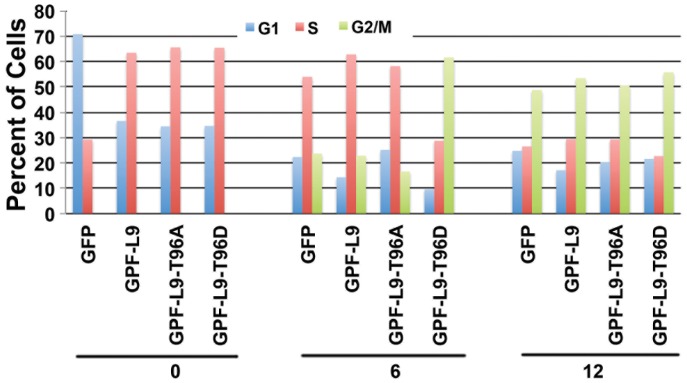
Phosphorylation of Thr-96 in LIN-9 accelerates entry into G2/M. 293T cells were transfected with the indicated LIN-9 constructs or GFP control and 24 hours later synchronized by serum starvation. Cells were released into the cell cycle by the addition of complete growth medium and harvested at 0, 6 and 12 hours for FACS analysis.

To investigate whether the observed transcriptional activation of cyclins A2, B1 and cdc6 by LIN9 and LIN9^T96D^ (but not LIN9^T96A^) results in accelerated cell cycle progression, we monitored the cell cycle distribution of 293T cells by FACS. Cells were transfected with the indicated GFP constructs, synchronized by serum starvation, and subsequently released into the cell cycle by serum addition. When cells were analyzed for their cell cycle distribution after transfection of different GFP-LIN-9 constructs, it became evident that transfection of LIN-9 (regardless of Thr96 status) allowed for cells to start synthesis of DNA as indicated by the notion that in cells transfected with GFP alone, most cells were efficiently arrested in G0/G1, whereas cells transfected with GFP-LIN-9 wild type, T96A, or T96D depicted an increased number of cells in S-phase (Figure 5 and Figure S1, G1, 0 hours). Once cells were released into the cell cycle by serum addition, the number of cells in G0/G1 phase rapidly decreased in cells transfected with GFP. More importantly, a very large percentage of cells transfected with LIN-9^T96D^ had already entered G2/M (61.6%) 6 hr after release (Fig. 5) as compared to cells transfected with GFP and GFP-LIN-9^wt^ (23.7 and 22.8%, respectively). Additionally, the number of cells in G2/M was reduced when cells were expressing GFP-LIN-9^T96A^ (16.6%) as compared to GFP control cells (23.7%) even though before serum release, most LIN-9-T96A expressing cells had already entered S-phase, suggesting an extended S-phase duration. Although twelve hours after release the cell cycle distribution was similar in all cells, 293T cells expressing the T96D mutant still showed a slightly increased number of cells in G2/M. These data strongly suggest that phosphorylation of T96 in LIN-9 triggers cell cycle progression likely through the transcriptional activation of specific cell cycle promoting genes.

## Discussion

Recently, a study employing phospho-mass-spectrometry demonstrated that a small fraction of LIN-9 is phosphorylated on Thr-112, a site corresponding to Thr-96 in our system, depending on the ATG considered as start site [24]. The finding of Thr-96 (or Thr-112 respectively) being phosphorylated in vivo strongly supports our notion of Thr-96 being an important phosphorylation site. However, neither the timing nor the kinase responsible for this phosphorylation or the biological relevance of this post-translational modification has been elucidated. In this report, we show that cyclin E/Cdk3 is the kinase responsible for phosphorylation of Thr-96 in LIN-9. Moreover, LIN-9 can be found in a complex with Cdk3 and cyclin E1. All these data strongly suggest that cyclin E/Cdk3 is responsible for the phosphorylation of Thr-96, a site that is phosphorylated in vivo (Litovchick et al., 2011). Thr-96 is also highly conserved among species indicating that this phosphorylation has biological relevance in vertebrates. The finding that LIN-9 phosphomimetic mutant proteins greatly activate promoters of known LIN-9 targets such as cdc6, cyclin B1 and cyclin A2, indicates that phosphorylation of Thr-96 in LIN-9 is a key event for transcriptional activation of cell cycle promoting genes. Concomitantly, expression of LIN-9^T96A^ inhibited the activation of LIN-9 target promoters below control levels, suggesting a dominant-negative action of this construct. Thus, LIN-9 phosphorylation on Thr-96 is an essential step for the induction of cdc6 and cyclins A2 and B1 and possibly other cell cycle genes. The strong activation of transcription of cdc6, cyclin B1 and A2 promoters is even more dramatic when taking into account that this form of LIN-9 is expressed at much lower levels than its wild type counterpart and the alanine mutant. We hypothesize that the induction of cdc6, cyclins A2 and B1 promoters translates into an accelerated cell cycle progression. In line with this notion, we found expression of the LIN-9^T96D^ mutant triggered massive entry of cells into G2/M phase as early as 6 hours after release from quiescence. These results indicate that cyclin E1/Cdk3 targets Thr-96 in LIN-9 for phosphorylation and this event triggers activation of cell cycle promoting genes such as cdc6, cyclin A2 and cyclin B1 and promotes S-phase progression and entry into G2/M phase. Interestingly, overexpression of all forms of LIN-9, wild type and mutants, impair the synchronization of 293T cells in G0/G1 suggesting that phosphorylation of LIN-9 – while being essential for S-phase progression – is dispensable for G0/G1 transition, at least in 293T cells. Importantly, the decrease in p-LIN-9^Thr96^ phosphorylation observed at the G1/S boundary, when LIN-9 is already bound to G2/M promoters [21], suggest that the mechanism of action of p-LIN-9^Thr96^ may be more complex than the simple activation of target genes within the context of the DREAM complex. For example, phosphorylation of LIN-9 on Thr^96^ may stabilize the complex on the promoter or recruit other transcriptional players. This would explain why LIN-9 is associated with the Cyclin A and B promoters before B-Myb and prior to their transcriptional activation [21].

In summary, we provide novel insights into the mechanism by which cyclin E controls the expression of subsequent cyclins through LIN-9. Furthermore, our findings suggest a novel mechanism by which cyclin E/Cdk3 promotes cell cycle transitions beyond G1/S phase and into S/G2 phase by triggering the expression of subsequent cyclins A and B through LIN-9. These findings are important because they elucidate the mechanism by which cyclin E passes on the torch to cyclins A and B, to promote timely cell cycle transitions.

## Materials and Methods

### Cell culture

T98G and 293FT cells (obtained from ATCC and Life Technologies, respectively) were grown in DMEM containing 10% FBS and 2 mM L-glutamine at 37°C with 5% CO_2_. HUVECs and endothelial cell growth medium were purchased from Lonza (Walkersville, MD). For synchronization, cells were serum starved for 36 hours and released into the cell cycle by addition of 10% FBS. Cells were transfected using Turbofect (Thermo Scientific) according to the manufacturer's instructions.

### Constructs and protein purification

GFP-LIN-9, GST-LIN-9 deletion constructs, and luciferase-constructs under the control of the promoters of cdc6, cyclin B1 or cyclin A2 have already been described [21], [28]. Untagged pCMV-cyclin E1 and pCMV-cyclin A2 constructs were a gift from Dr. P Kaldis. Flag-Cdk3 and Flag-LIN-9 constructs were purchased from GeneCopoeia. GST- and GFP-LIN-9 mutants, and Flag-Cdk3-DN were generated by site-directed mutagenesis as described previously [29]. Expression, purification and dialysis of GST-proteins have been described previously [30].

### Antibodies and immunoprecipitation

Antibodies for LIN-9 have been described previously [26] and the anti-phosphoT96 was developed by ProteinTech, Inc (Chicago, IL). Antibodies for cyclin E1, cyclin A2 and GFP were from Santa Cruz, antibody for Flag was from Sigma. For IP, cells were lysed in NP-40 lysis buffer (300 mM NaCl, 50 mM Tris pH 8.0, 1 mM MgCl_2_, 0.2% NP-40, 10% Glycerol, Protease- and Phosphatase-Inhibitors from Roche and Sigma, respectively). For Co-IPs, cell lysis was performed in NP-40 lysis buffer, followed by IP in TIF-buffer (150 mM NaCl, 20 mM Tris pH 8.0, 1 mM MgCl_2_, 0.1% NP-40, 10% Glycerol and Protease- and Phosphatase-inhibitors from Roche and Sigma, respectively).

### Kinase assays and lambda phosphatase assay

Kinase assays using both recombinant kinases from ProQinase (Freiburg, Germany) or Flag-Cdk3 kinase complexes, immunoprecipitated from 293T cells, were performed as described previously [31] with the exception, that kinase reactions were performed in a kinase buffer containing 60 mM HEPES pH7.5, 3 mM MgCl_2_, 3 mM MnCl_2_, 1.2 mM DTT, 100 cold µM ATP, 2 µCi of [γ-^32^P]ATP at 30°C for 10 min, if not indicated otherwise. For lambda phosphatase assay, 200 U of lambda phosphatase (NEB) were user per IP in 1× NEBuffer for PMP (NEB) supplemented with 1 mM MnCl_2_ for 1 hour at 30°C according to the manufacturer's instructions.

### Reporter gene assays

Reporter gene assays were performed as described previously [32], with the exception that cells were plated and transfected on 6 well dishes and cell lysis and analysis was performed using the Promega Glo (Promega) system, following the manufacturer's instructions.

## Supporting Information

Figure S1 **FACS histograms of the data described in** **Figure 5** **.**
(TIF)
