# Supplementary figures and images for: LIN-9 Phosphorylation on Threonine-96 Is Required for Transcriptional Activation of LIN-9 Target Genes and Promotes Cell Cycle Progression
Source: PLoS One. 2014 Jan 27;9(1):e87620. doi: 10.1371/journal.pone.0087620 (PMC3903767; doi:10.1371/journal.pone.0087620)

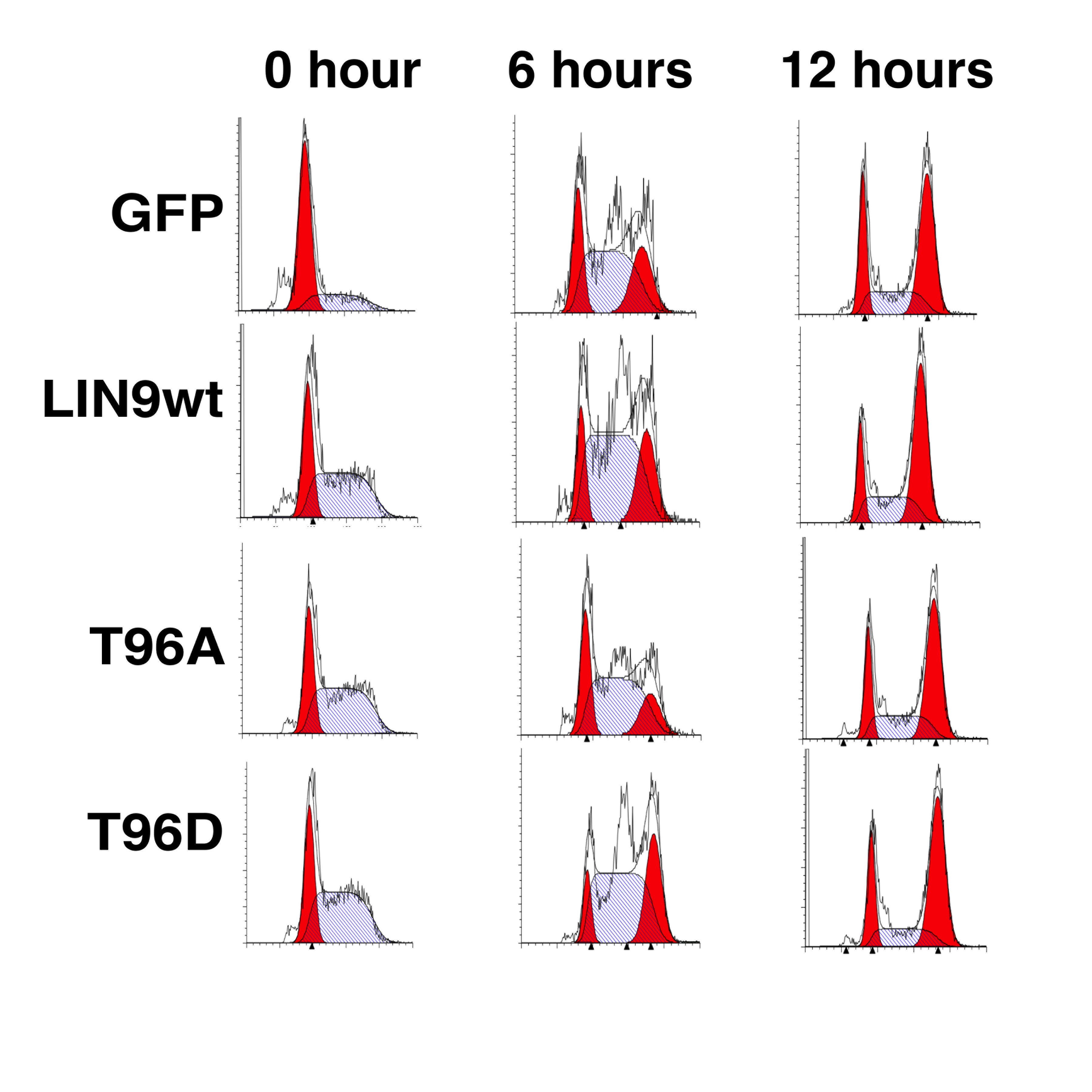

Supplement: Figure S1 — FACS histograms of the data described in Figure 5 . (TIF) [file pone.0087620.s001.tif]
